# Supplementary material for: Generalised optical printing of photocurable metal chalcogenides
Source: Nat Commun. 2022 Sep 7;13:5262. doi: 10.1038/s41467-022-33040-2 (PMC9452581; doi:10.1038/s41467-022-33040-2)
Supplement: Supplementary file 1 — Supplementary Information [file 41467_2022_33040_MOESM1_ESM.pdf]

Generalised optical printing of photocurable metal chalcogenides

Seongheon Baek[1†], Hyeong Woo Ban[1†], Sanggyun Jeong[2†], Seung Hwae Heo[1], Da Hwi Gu[1], Wooyong Choi[1], Seungjun Choo[1], Yae Eun Park[3], Jisu Yoo[1], Moon Kee Choi[1,3,4] and Jiseok Lee[2]*, & Jae Sung Son[1,3]*

[1]*Department of Materials Science and Engineering, Ulsan National Institute of Science and Technology (UNIST), Ulsan 44919, Republic of Korea*

[2]*Department of Energy Engineering, School of Energy and Chemical Engineering, Ulsan National Institute of Science and Technology (UNIST), Ulsan, Republic of Korea*

[3]*Graduate School of Semiconductor Materials and Devices, Ulsan National Institute of Science and Technology (UNIST), Ulsan 44919, Republic of Korea*

[4]*Center for Nanoparticle Research, Institute for Basic Science (IBS), Seoul 08826, Republic of Korea*

[†]*These authors contributed equally to this work.*

Correspondences to: jiseok@unist.ac.kr (J.L.) and jsson@unist.ac.kr (J.S.S.)

This supplement contains

Supplementary Figure 1-20

Supplementary Table 1-3

Supplementary Discussion

Supplementary References

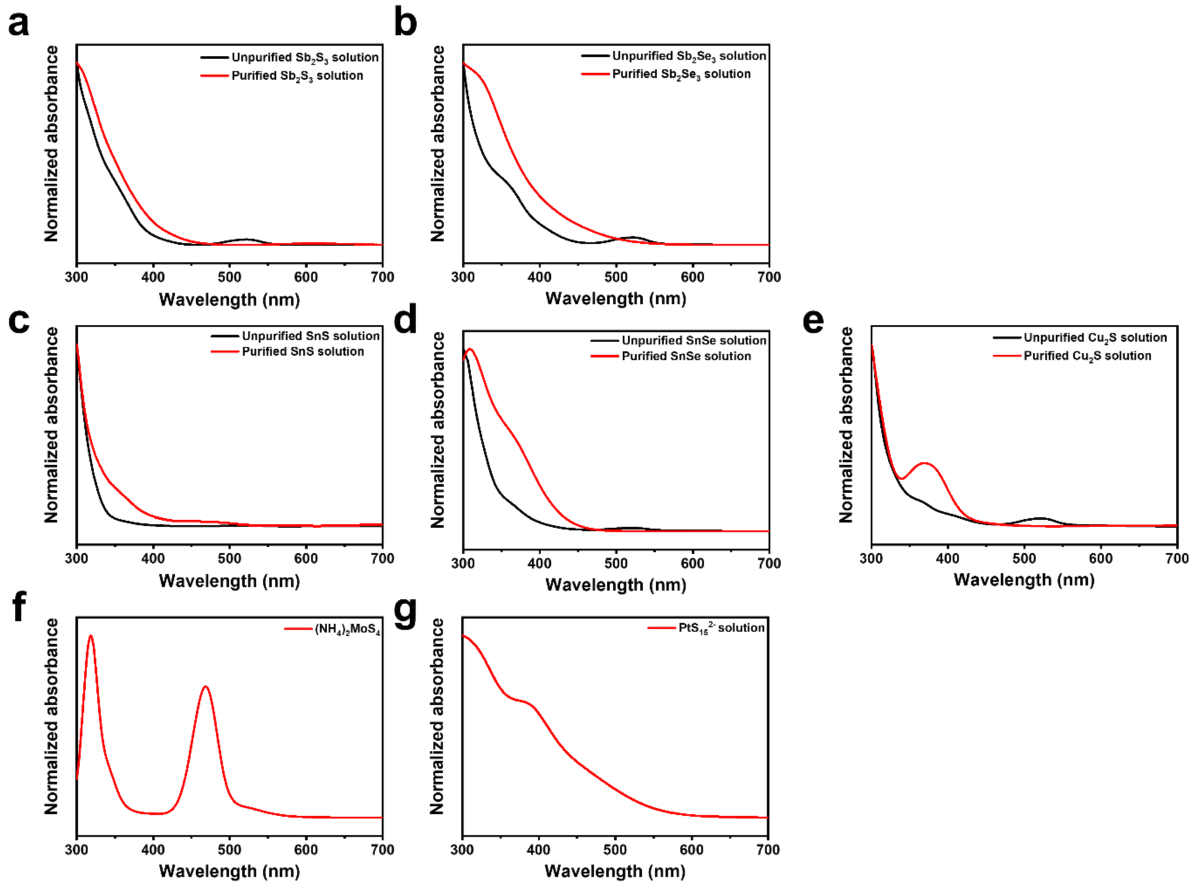

Supplementary Figure 1 | UV-Vis absorption spectrum of (**a**) Sb_2S_3-, (**b**) Sb_2Se_3-, (**c**) SnS-, (**d**) SnSe-, (**e**) Cu_2S-, (**f**) MoS_2-, and (**g**) PtS_2-based ChaM inks.

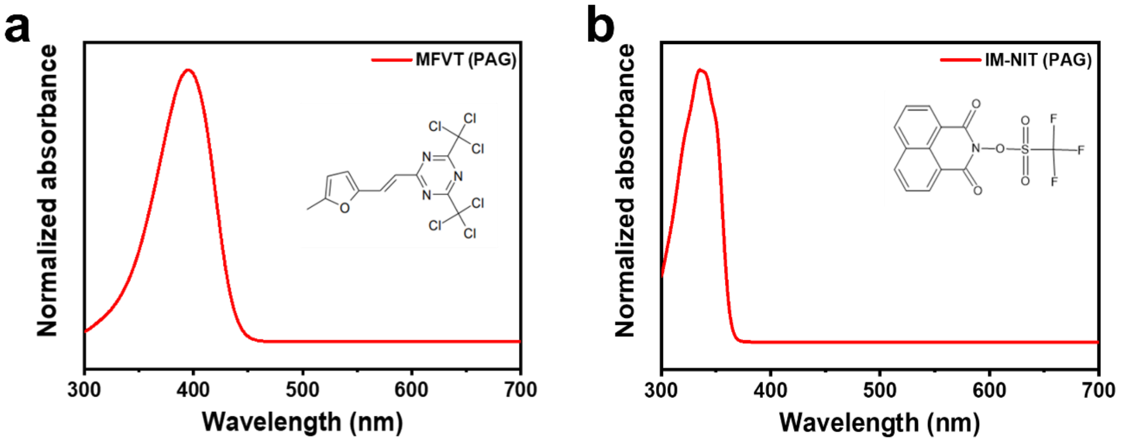

Supplementary Figure 2 | UV-Vis absorption spectrum and chemical structure of PAG (**a**) MFVT and (**b**) IM-NIT.

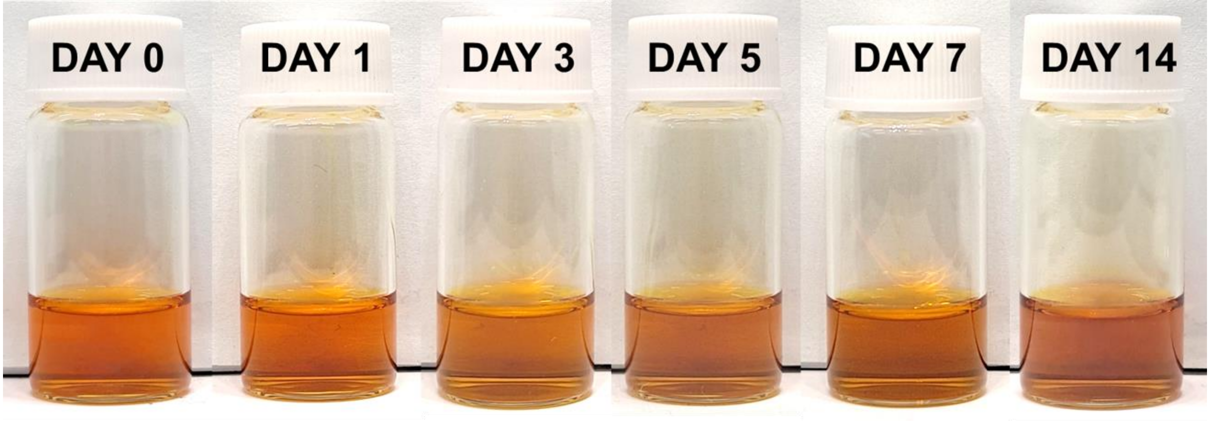

Supplementary Figure 3 | Photographs showing the time-evolution stability of photocurable PtS_2-based inks.

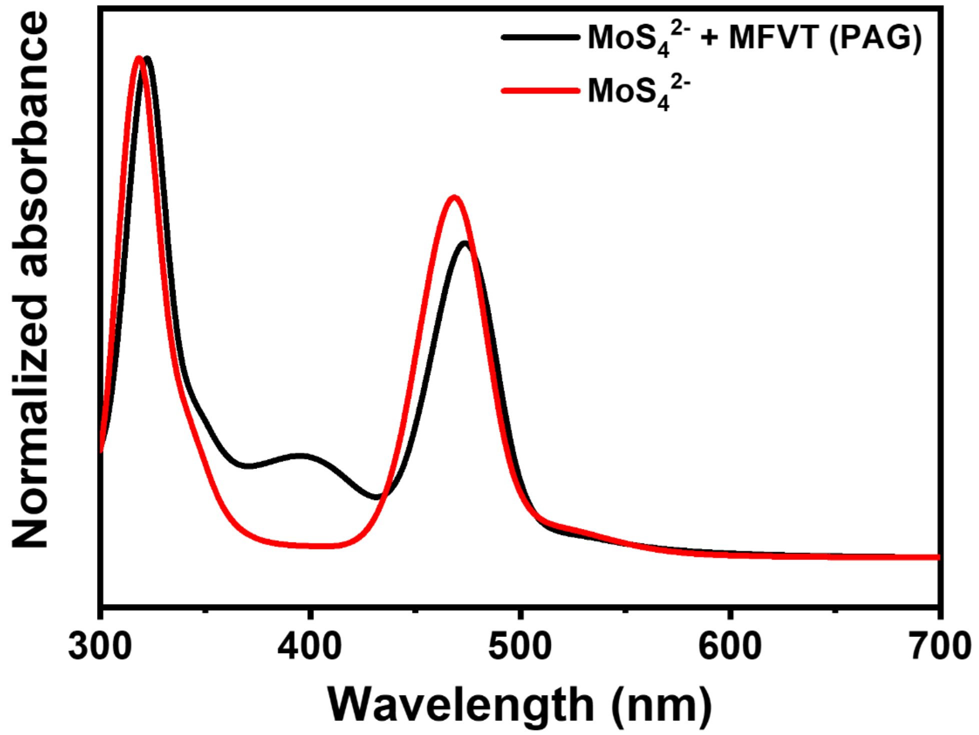

Supplementary Figure 4 | UV-Vis absorption spectrum of photocurable MoS_2-based inks and MoS_4^{2-} ChaM solution.

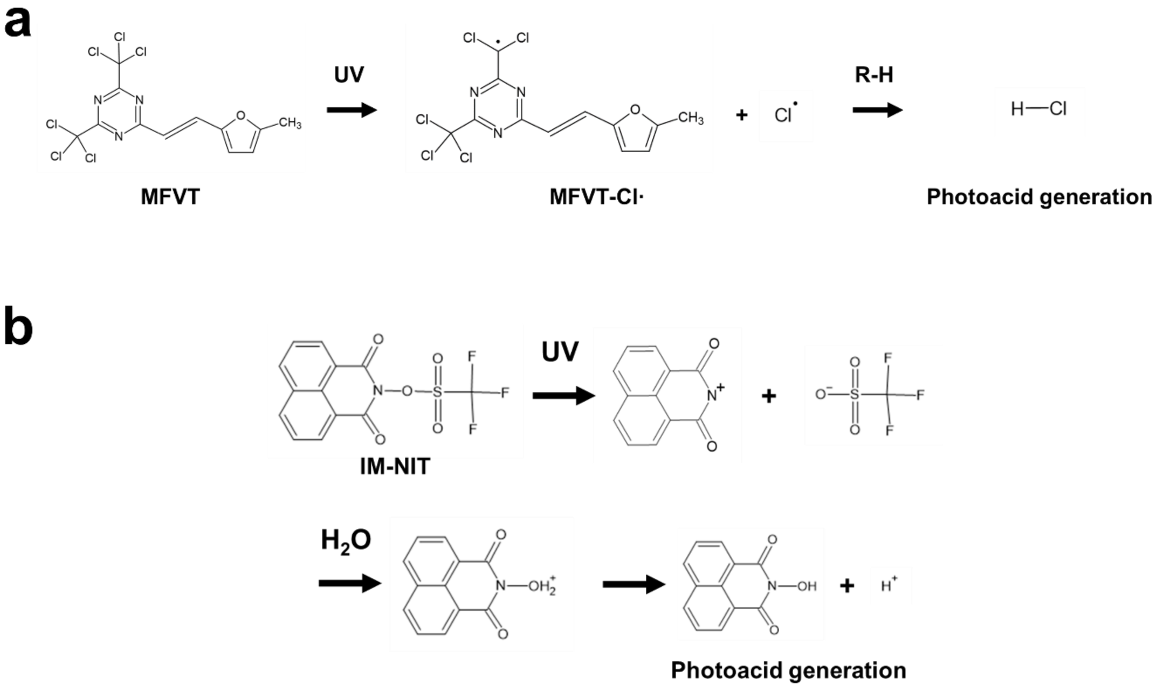

Supplementary Figure 5 | The chemical reaction of photoacid generation (**a**) MFVT and (**b**) IM-NIT.

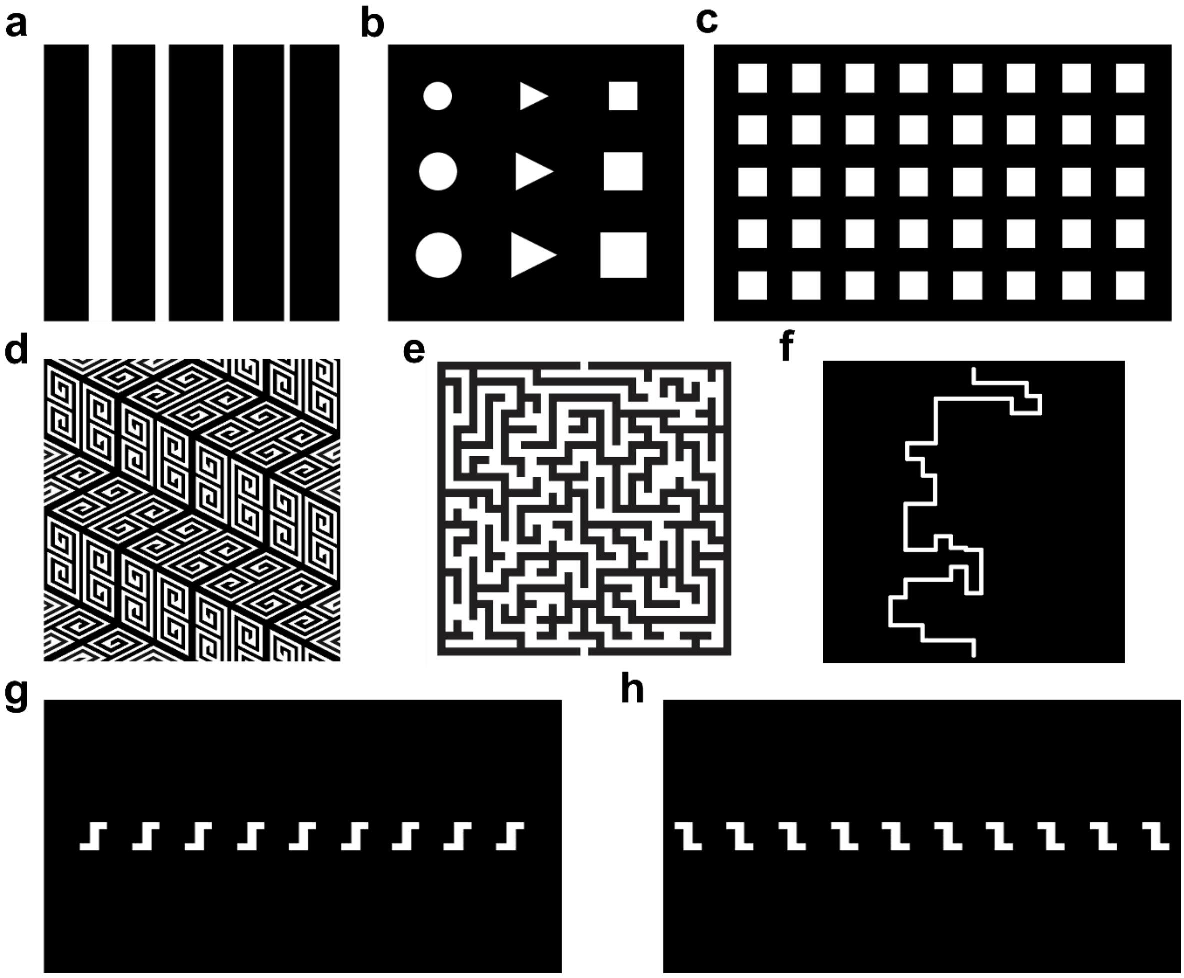

Supplementary Figure 6 | Designed digital masks for the DLP printing. (**a**) lines, (**b**) shapes, (**c**) multiple squares, (**d**) geometric pattern, (**e**) maze, (**f**) solving line, and thermoelectric (**g**) n-type and (**h**) p-type legs.

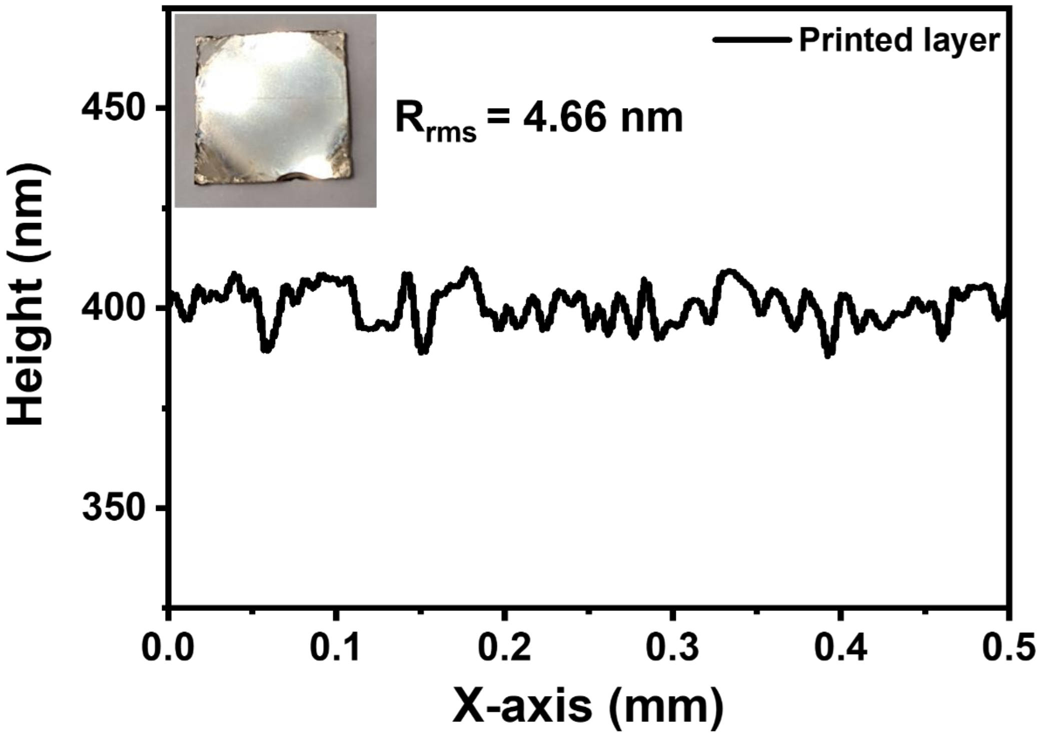

Supplementary Figure 7 | Root-mean-square surface roughness of the printed layer.

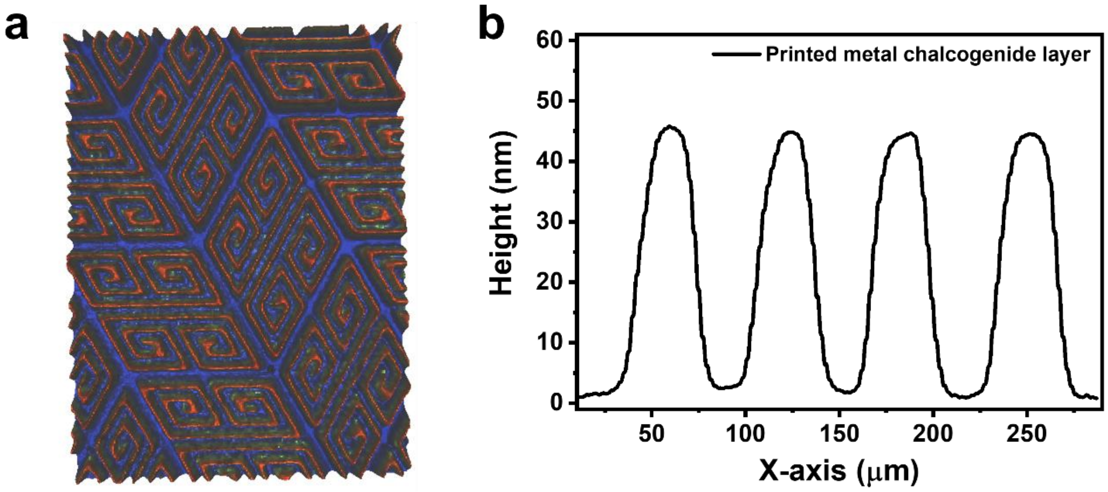

Supplementary Figure 8 | (**a**) Interferometric scattering-based 3D scanner images of geometric patterns and (**b**) height profiles.

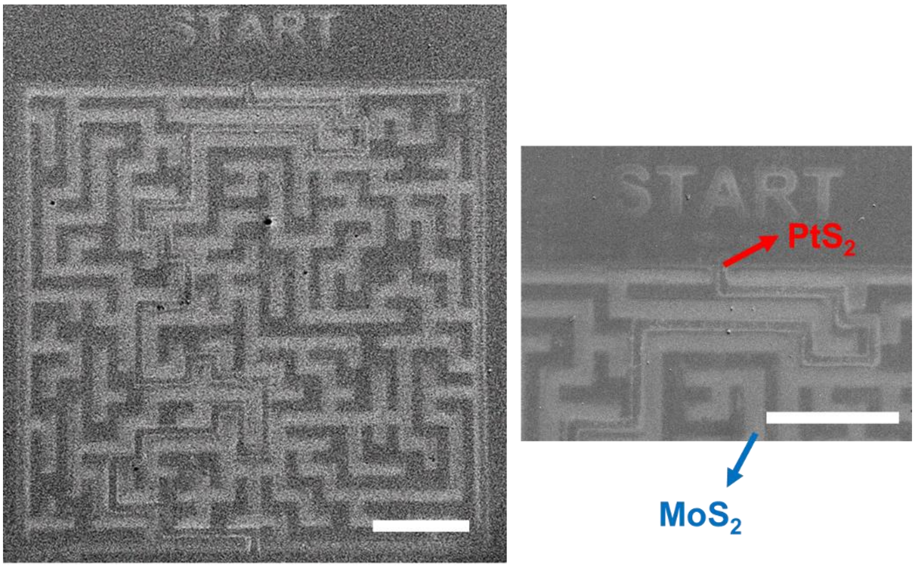

Supplementary Figure 9 | SEM images of the printed maze and its solution line patterned with multiple ChaMs. (Scale bar: 500 μm)

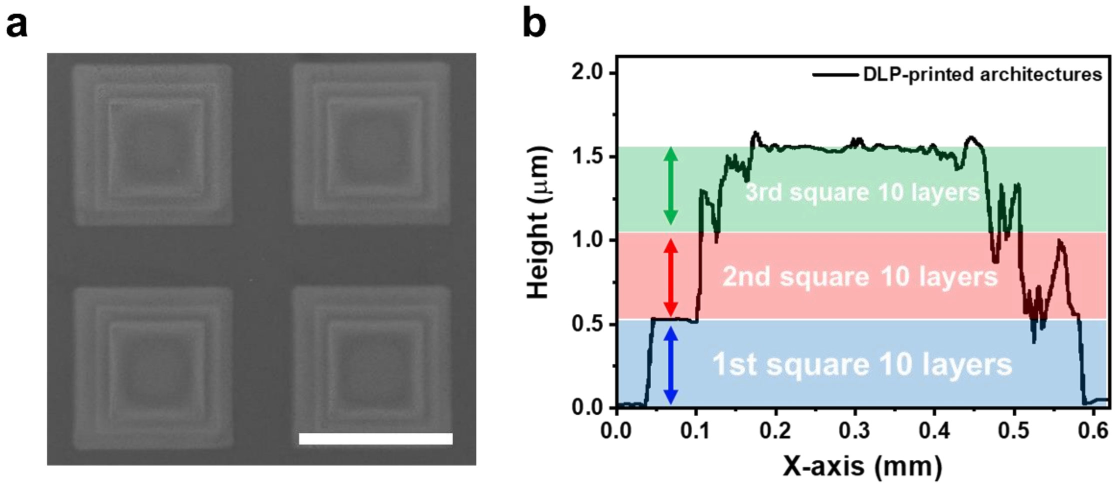

Supplementary Figure 10 | (a) SEM image of the DLP-printed pyramidal architectures (b) Height profile of the DLP-printed architectures. (Scale bar: 500 μm)

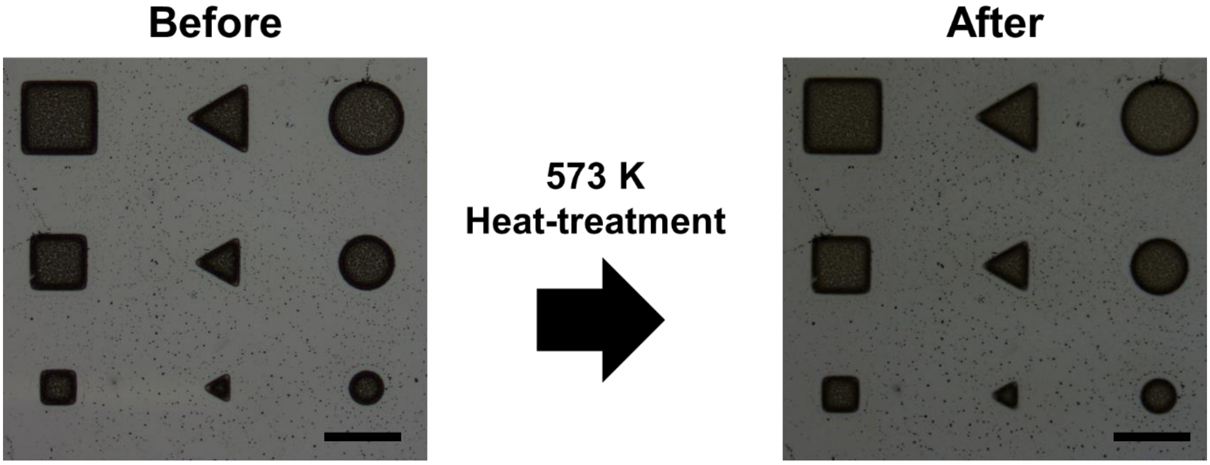

Supplementary Figure 11 | Optical microscope images of the printed structures before/after heat-treatment. (Scale bar: 200 µm)

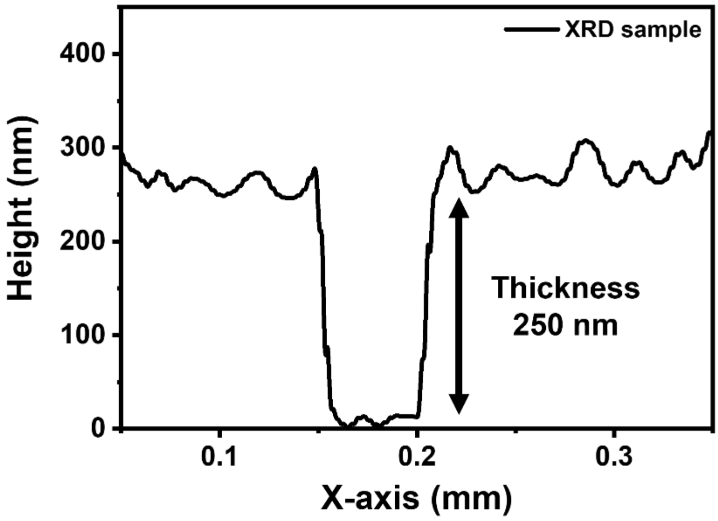

Supplementary Figure 12 | 3D scan height profile of the printed samples for XRD analysis.

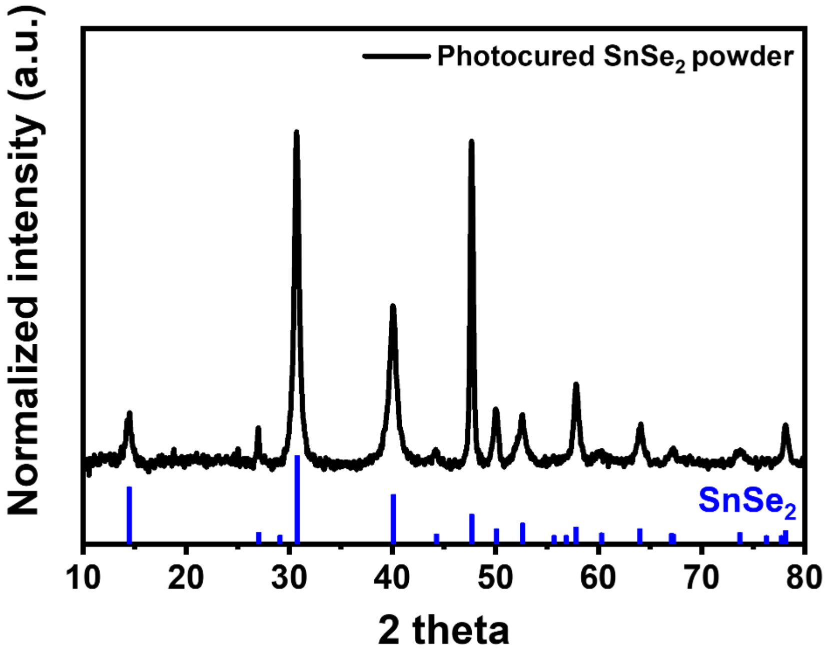

Supplementary Figure 13 | XRD pattern of photocured $SnSe_2$ powder.

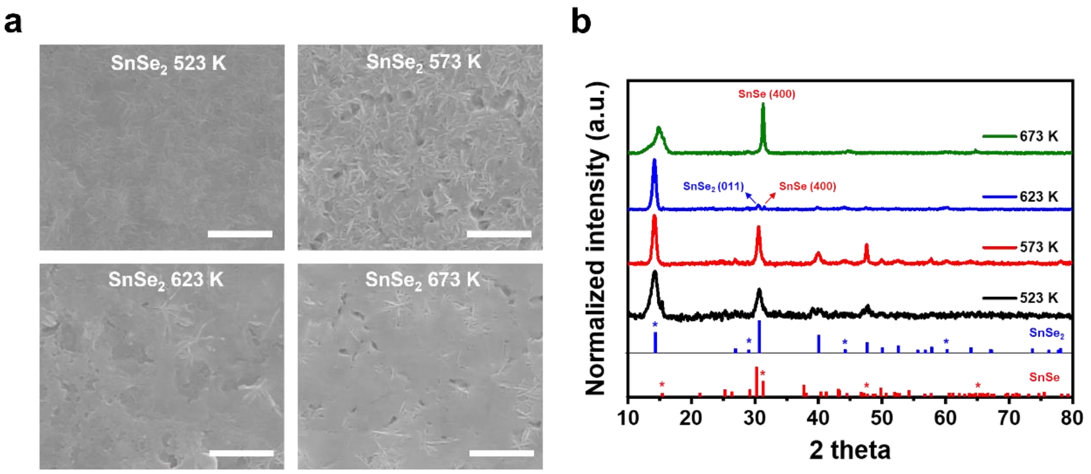

Supplementary Figure 14 | (a) SEM images and (b) XRD patterns of the printed $SnSe_2$ according to the heat treatment temperature.

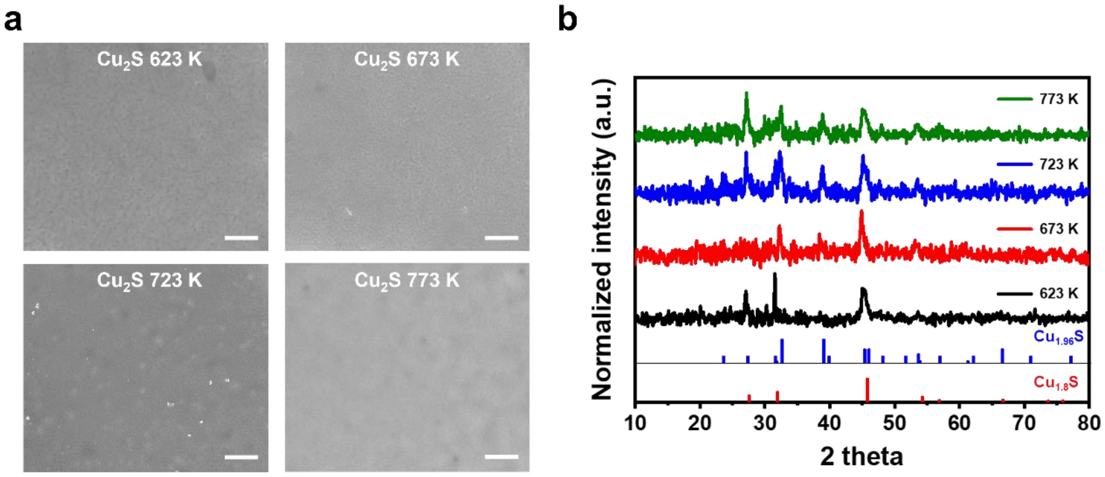

Supplementary Figure 15 | (a) SEM images and (b) XRD patterns of the printed Cu_2S according to the heat treatment temperature.

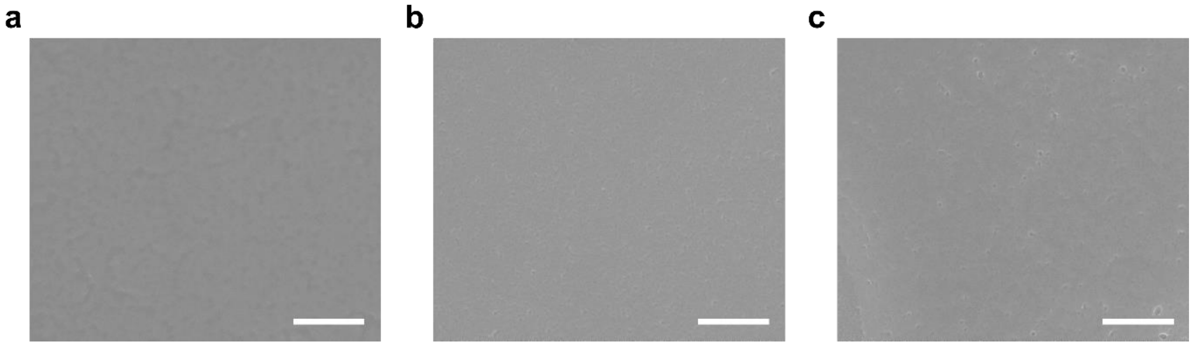

Supplementary Figure 16 | SEM images of the printed Cu_2S layer annealed for (**a**) 5 min, (**b**) 10 min and (**c**) 15 min at 723 K. (Scale bar: 2 μm)

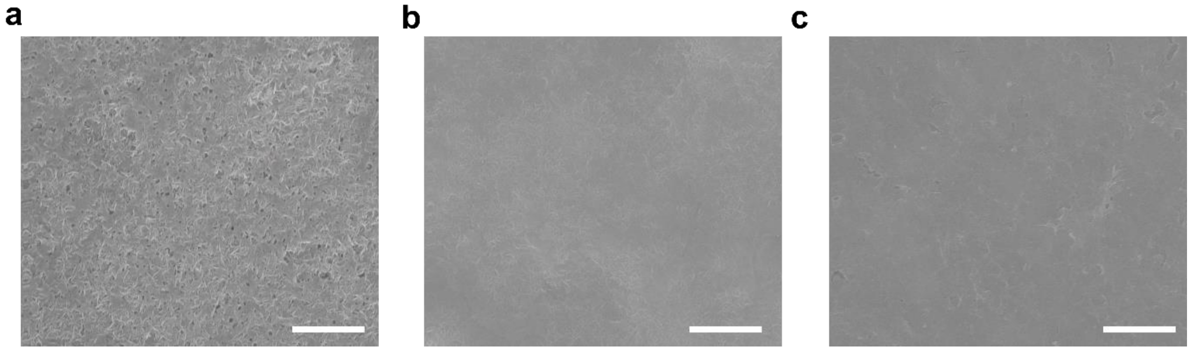

Supplementary Figure 17 | SEM images of the printed $SnSe_2$ layer annealed for (**a**) 7 min, (**b**) 10 min and (**c**) 15 min at 573 K. (Scale bar: 2 μm)

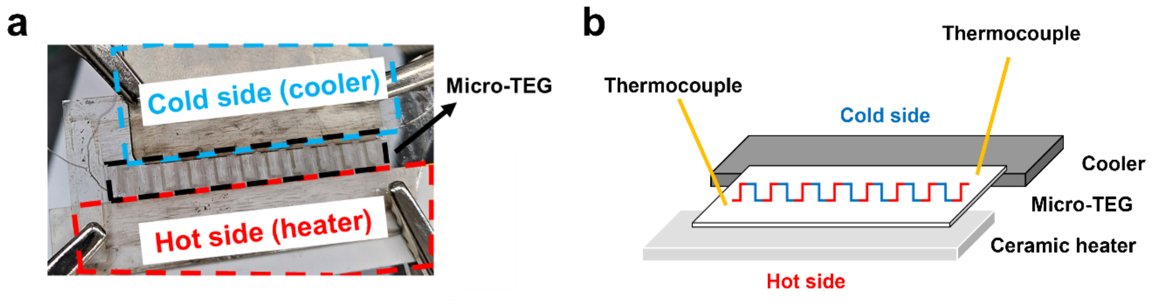

Supplementary Figure 18 | (**a**) Photographs of the fabricated micro thermoelectric generator and (**b**) schematic image.

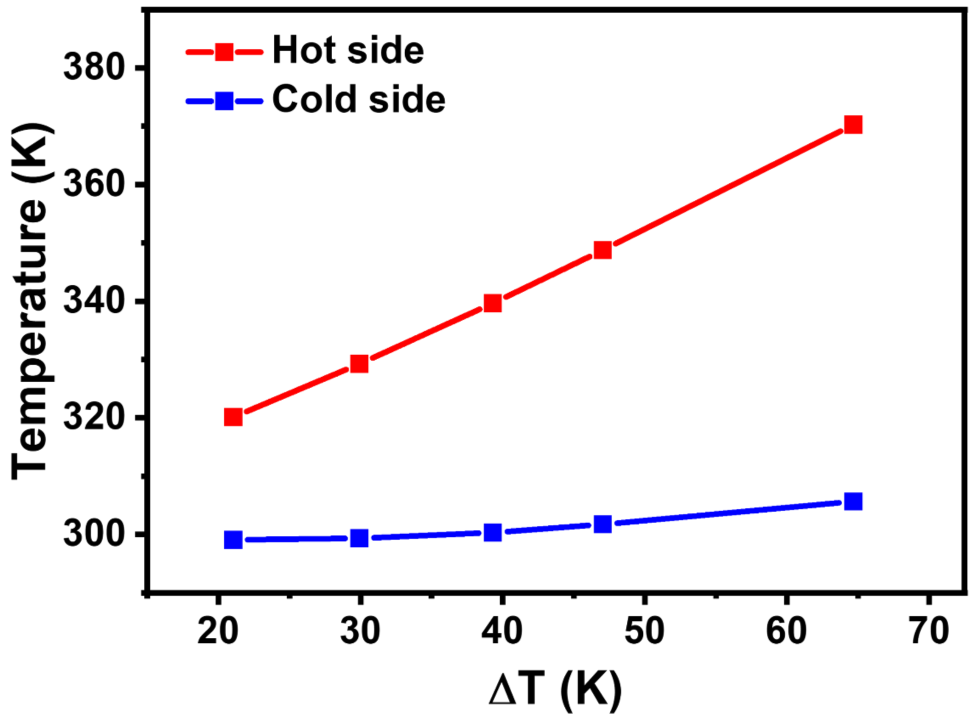

Supplementary Figure 19 | Temperatures of the hot and cold sides of the micro thermoelectric generator measuring power performance under heating up.

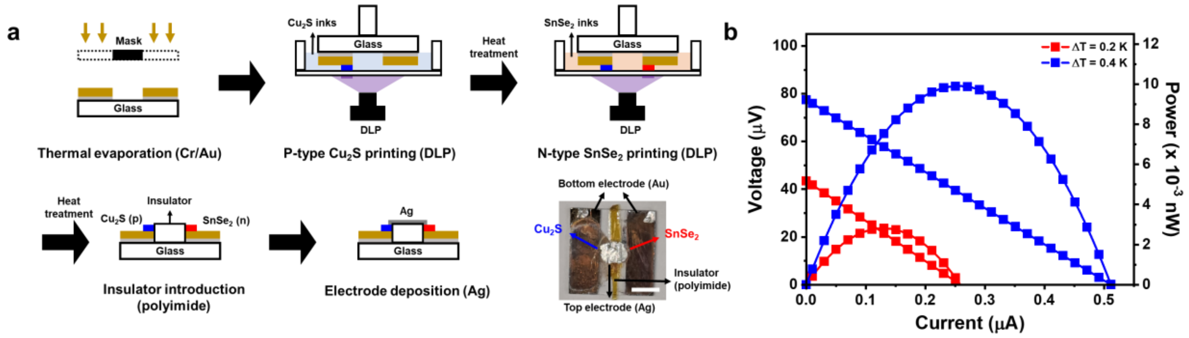

Supplementary Figure 20 | (**a**) Scheme for the fabrication of the cross-plane thermoelectric generator consisting of the DLP-printed Cu_2S and $SnSe_2$ semiconductor layers (scale bar: 5 mm). (**b**) Output voltages and powers of the fabricated cross-plane thermoelectric generator.

Supplementary Table. 1 | Comparison of mobility with metal chalcogenide and inorganic patterns fabricated by lithographic techniques or other printing methods.

Materials	Mobility ($cm^2\ V^{-1}\ s^{-1}$)	Processing	Reference
PbS	1.8	Spin coating and direct patterning (Electron beam lithography)	[1]
GaS	0.2	Photolithography and printing the skin oxide of liquid Ga	[2]
PbSe, CdSe	$1.0 \pm 0.3 \times 10^{-1}$ (PbSe)<br>1.5 ± 0.5 (CdSe)	Ink-lithography	[3]
In-Ga-Zn Oxide (IGZO)	4.2	Spin coating and direct patterning (UV irradiation using photomask)	[4]
CuI	1.86 ± 1.5	Inkjet printing	[5]
CdSe, IGZO	20 to 109 (CdSe)<br>4 to 10 (IGZO)	Direct optical lithography of functional inorganic nanomaterial (DOLFIN)	[6]
$CuInSe_2$	0.69 (n-type)<br>3.18×10^{-3} (p-type)	Spin coating on a pre-patterned substrate (shadow mask thermal evaporation)	[7]
Cu_2S, $SnSe_2$	3.89 (Cu_2S)<br>2.25 ($SnSe_2$)	Direct optical printing from inks by DLP printing	Our work

Supplementary Table. 2 | Comparison of the printed materials in this study with the corresponding reported materials fabricated by the different methods

Ref	Materials	Properties (*n*: carrier concentration<br>*μ*: carrier mobility<br>*σ*: electrical conductivity)	Sample type and fabrication method
[8]	$Cu_{2-x}S$	*n*: 7.27×10^{20} cm^{-3} at RT ($Cu_{1.97}S$)<br>*μ*: 0.69 cm^2 V^{-1} S^{-1} at RT ($Cu_{1.97}S$)<br>*σ*: 80 S cm^{-1} at RT	Bulk $Cu_{1.97}S$ pellets obtained by spark plasma sintering
[9]	$Cu_{2-x}S$	*n*: 7.92×10^{19} cm^{-3} at RT ($Cu_2S_{0.9}Se_{0.1}$)<br>*μ*: 6.02 cm^2 V^{-1} S^{-1} at 300 K ($Cu_2S_{0.9}Se_{0.1}$)<br>*σ*: 56.78 S cm^{-1} at RT	Bulk $Cu_2S_{0.9}Se_{0.1}$ pellets obtained by spark plasma sintering
[10]	$Cu_{2-x}S$	*n*: ~ 10^{19} cm^{-3} at RT<br>*μ*: 4.28 cm^2 V^{-1} S^{-1} (spin-coating)<br>*σ*<br>75 S cm^{-1} at RT (EPD)<br>5.7 S cm^{-1} at RT (spin-coating)	$Cu_{1.94\text{-}1.96}S$ nanoparticles thin films obtained by electrophoretic deposition (EPD) and spin-coating
[11]	$Cu_{2-x}S$	*n*: N/A<br>*μ*: N/A<br>*σ*: 20.25 S cm^{-1} at RT	Pseudo-3D printed $Cu_{1.97}S$ bulk
This work	$Cu_{2-x}S$	*n*: 6.29×10^{19} cm^{-3} at RT<br>*μ*: 3.89 cm^2 V^{-1} s^{-1}<br>*σ*: 10 S cm^{-1} at RT	DLP-printed $Cu_{2-x}S$ film
[12]	$SnSe_2$	*n*: 2.26×10^{18} cm^{-3} at RT<br>*μ*<br>31.6 cm^2 V^{-1} S^{-1} (ab plane) at RT<br>13.2 cm^2 V^{-1} S^{-1} (c axis) at RT<br>*σ*<br>11.4 S cm^{-1} (ab plane) at RT<br>4.8 S cm^{-1} (c axis) at RT	$SnSe_2$ single crystals synthesised by a temperature gradient method.
[13]	$SnSe_2$	*n*: 8.4×10^{17} cm^{-3} at RT<br>*μ*: N/A<br>*σ*: ~2 S cm^{-1} at 350 K (cross plane)	Textured $SnSe_2$ nanostructured bulk materials obtained by hot-pressing of $SnSe_2$ nanoplates
[14]	$SnSe_2$	*n*: N/A<br>*μ*: N/A<br>*σ*: ~ 10 S cm^{-1} at RT	$SnSe_2$ thin film fabricated by spin-coating of $SnSe_2$ nanocrystals
This work	$SnSe_2$	*n*: ~ 10^{19} cm^{-3} at RT<br>*μ*: 2.25 cm^2 V^{-1} s^{-1}<br>*σ*: 2.79 S cm^{-1} at RT	DLP-printed $SnSe_2$ film

Supplementary Table. 3 | Comparison of the current optical printing method with the recent state-of-the-art printing methods for inorganics.

Printing method [ref]	Printable materials	Printing speed and resolution	Printing steps	**Properties**<br>(ρ: resistivity<br>R: resistance<br>μ: carrier mobility<br>σ: electrical conductivity<br>S: Seebeck coefficient)	Remark
Direct patterning [1]	Metal chalcogenides	Printing speed<br>~ 2 h (exposure)<br><br>Resolution<br>~ 50 nm	Three steps (Coating, exposure and developing)	Sb_2S_3 ρ: 2.7 × 10^5 Ω m<br>PbS μ: 1.8 cm^2 V^{-1} S^{-1}	Several lithographic technologies are applicable (Electron beam lithography, two-photon absorption lithography, thermal scanning probe lithography, UV light lithography)
Direct optical lithography [6]	Metal, semiconductor, oxide nanocrystals	Printing speed<br>Tens of second (exposure)<br><br>Resolution<br>~ 1 μm	Three steps (Coating, exposure and developing)	Au ρ: 5.2 × 10^{-8} Ω m<br>CdSe μ: 20 ~ 100 cm^2 V^{-1} S^{-1}<br>IGZO μ: 4 ~ 10 cm^2 V^{-1} S^{-1}	Direct photolithography of spin-coated nanocrystal film
Light-induced material deposition (laser writing) [15]	Metals and insulator (Iron oxide)	Printing speed<br>Exposure time per pixel 30 ms ~ 1 s,<br>Pixel size<br>446 nm ~ 1.17μm<br><br>Resolution<br>500 nm ~ 1 μm	Two steps (Exposure and cleaning)	Pt R: 12.8 Ω<br>(18.8% of bulk)<br>Ni R <100 Ω<br>Zn R >10 kΩ<br>Fe R >200 MΩ<br>Au R >200 MΩ	Laser writing on the drop-casted metallate solution
Ink lithography (Ink-jet printing) [3]	Metal, semiconductor, oxide, perovskite nanocrystals	Printing speed<br>300 mm s^{-1}<br><br>Resolution<br>~ 35μm	Three steps (Coating, printing, and developing)	Ag resistivity: 3.2±1.1 ×10^{-5} Ω m<br>PbSe μ:1.0±0.3 × 10^{-1} cm^2 V^{-1} S^{-1}<br>CdSe μ:1.5±0.5 cm^2 V^{-1} S^{-1}	Inkjet printing, pen writing or brush painting are applicable<br>Ligand inks printed on spin-coated nanocrystal film.
Ink-jet printing [16]	Perovskite	Printing speed<br>Thousands of seconds<br><br>Resolution<br>~ 100 μm	Three steps (Coating, printing, and drying)	Photoluminescence quantum yield ~ 80%	Variation of pattern size by nozzle and drying temperature<br>Printed on the polymer film
Transfer printing [17]	Quantum dot nanocrystals	Printing speed<br>Pick-up 10 cm s^{-1}<br>Detach < 1 mm s^{-1}<br><br>Resolution<br>~ 1 μm	Five steps (Coating, pick-up, contact, detach, and transfer)	Electroluminescence performance<br>14,000 cd m^{-2} at 7 V<br><br>Quantum yield > 80%	Intaglio transfer printing.<br>Need to fabricate a PDMS stamp and intaglio trench
Pseudo-3D printing [11]	$Cu_{2-x}S$	Printing speed<br>-<br><br>Resolution<br>Size of mould	Two steps (Moulding and pouring)	σ: 20.25 S cm^{-1} at RT<br>S: ~100 μV K^{-1} at 600 K	Need moulding apparatus

Extrusion-based 3D printing [18]	Cu_2Se	Printing speed 3 mm s^{-1}<br><br>Resolution ~ 330 μm	One step	*σ*: 183.42 S cm^{-1}<br>*S*: 185.4 μV K^{-1}	Topologically designed 3D printing for thermoelectric
Direct ink writing [19]	p: $Bi_{0.55}Sb_{1.45}Te_3$<br>n: $Bi_2Sb_{2.7}Se_{0.3}$	Printing speed 2 mm s^{-1}<br><br>Resolution ~ 180 μm	One step	*σ*: 650 (p), 793.2 (n) S cm^{-1}<br>*S*: 196.6 (p), -110.1(n) μV K^{-1}	Direct-written filaments-based micro-TE devices
This work (DLP-based optical printing)	Metal chalcogenides (ChaM-based inks)	**Printing speed** Few ~ tens second (exposure)<br><br>**Resolution** ~ 25 μm	**Two steps** (Printing and rinsing)	P-type Cu_2S<br>*μ*: 3.89 cm^2 V^{-1} s^{-1}<br>*σ*: 10 S cm^{-1} at RT<br>*S*: 87 μV K^{-1} at RT<br><br>N-type $SnSe_2$<br>*μ*: 2.25 cm^2 V^{-1} s^{-1}<br>*σ*: 2.79 S cm^{-1} at RT<br>*S*: -203 μV K^{-1} at RT	Directly printing from inks<br>Need no photomask or mould<br>High-throughput printing at once<br>Compatibility with 3D printing

Supplementary Discussion

To optimise the annealing conditions, we systematically annealed the $SnSe_2$ and Cu_2S samples at different temperatures and characterised them by the XRD and SEM analysis. The SEM images of $SnSe_2$ samples (Supplementary Figure 14) shows that the microstructures were not changed significantly. However, the SnSe phase (p-type) was started to detect in the XRD patterns at 623 K and the $SnSe_2$ phase was fully transformed to the SnSe phase at 673 K. This composition transition was well-known to be attributable to the evaporation of Se[20]. Since SnSe crystal generally exhibit the p-type properties, we chose the annealing temperature of 573 K to conserve the n-type character of the $SnSe_2$ sample. On the other hand, regardless of the annealing temperatures, all Cu_2S samples shows the smooth film with good coverage, as shown in the SEM images (Supplementary Figure 15). However, the XRD patterns of the samples annealed at 623 K and 673 K corresponded to the $Cu_{1.8}S$ bulk reference, while the samples annealed at higher temperatures showed the $Cu_{1.96}S$ phase (Supplementary Figure 15b). In general, $Cu_{1.96}S$ crystal is known to exhibit significantly higher thermoelectric properties than those of $Cu_{1.8}S$[21]. Accordingly, we chose the annealing temperature of 723 K to obtain the $Cu_{1.96}S$ phase.

Supplementary References

1. Wang, W., Pfeiffer, P. & Schmidt-Mende, L. Direct Patterning of Metal Chalcogenide Semiconductor Materials. *Adv. Funct. Mater.* **30**, 2002685 (2020).

2. Carey, B. J. *et al.* Wafer-scale two-dimensional semiconductors from printed oxide skin of liquid metals. *Nat. Commun.* **8**, 14482 (2017).

3. Ahn, J. *et al.* Ink-Lithography for Property Engineering and Patterning of Nanocrystal Thin Films. *ACS Nano* **15**, 15667-15675 (2021).

4. Miyakawa, M., Nakata, M., Tsuji, H. & Fujisaki, Y. Simple and reliable direct patterning method for carbon-free solution-processed metal oxide TFTs. *Sci. Rep.* **8**, 12825 (2018).

5. Choi, C.-H. *et al.* Low-temperature, inkjet printed p-type copper(i) iodide thin film transistors. *J. Mater. Chem. C* **4**, 10309-10314 (2016).

6. Wang, Y., Fedin, I., Zhang, H. & Talapin, D. V. Direct optical lithography of functional inorganic nanomaterials. *Science* **357**, 385-388 (2017).

7. Yun, H. J., Lim, J., Roh, J., Neo, D. C. J., Law, M. & Klimov, V. I. Solution-processable integrated CMOS circuits based on colloidal $CuInSe_2$ quantum dots. *Nat. Commun.* **11**, 5280 (2020).

8. He, Y. *et al.* High thermoelectric performance in non-toxic earth-abundant copper sulfide. *Adv. Mater.* **26**, 3974-3978 (2014).

9. Yao, Y., Zhang, B.-P., Pei, J., Liu, Y.-C & Li, J.-F. Thermoelectric performance enhancement of Cu_2S by Se doping leading to a simultaneous power factor increase and thermal conductivity reduction. *J. Mater. Chem. C.* **5**, 7845-7852 (2017).

10. Otelaja, O. O., Ha, D. H., Ly, T., Zhang, H. & Robinson, R. D. Highly conductive $Cu_{2-x}S$ nanoparticle films through room-temperature processing and an order of magnitude enhancement of conductivity via electrophoretic deposition. *ACS Appl. Mater. Interfaces* **6**, 18911-18920 (2014).

11. Burton, M. R., Mehraban, S., McGettrick, J., Watson, T., Lavery, N. P. & Carnie, M. J. Earth abundant, non-toxic, 3D printed $Cu_{2-x}S$ with high thermoelectric figure of merit. *J. Mater. Chem. A.* **7**, 25586-25592 (2019).

12. Pham, A.-T. *et al.* High-Quality $SnSe_2$ Single Crystals: Electronic and Thermoelectric Properties. *ACS Appl. Energy Mater.* **3**, 10787-10792 (2020).

13. Zhang, Y. *et al.* Tin Diselenide Molecular Precursor for Solution-Processable Thermoelectric Materials. *Angew. Chem.* **130**, 17309-17314 (2018).

14. Yin, D,, Liu, Y., Dun, C., Carroll, D. L. & Swihart, M. T. Controllable colloidal synthesis of anisotropic tin dichalcogenide nanocrystals for thin film thermoelectrics. *Nanoscale* **10**, 2533-2541 (2018).

15. Chen, Y. *et al.* A universal method for depositing patterned materials in situ. *Nat. Commun.* **11**, 5334 (2020).

16. Shi, L.,*et al.* In Situ Inkjet Printing Strategy for Fabricating Perovskite Quantum Dot Patterns. *Adv. Funct. Mater.* **29**, 1903648 (2019).

17. Choi, M. K. *et al.* Wearable red-green-blue quantum dot light-emitting diode array using high-resolution intaglio transfer printing. *Nat. Commun.* **6**, 7149 (2015).

18. Choo, S. *et al.* Cu_2Se-based thermoelectric cellular architectures for efficient and durable power generation. *Nat. Commun.* **12**, 3550 (2021).

19. Kim, F. *et al.* Direct ink writing of three-dimensional thermoelectric microarchitectures. *Nat. Electron.* **4**, 579-587 (2021).

20. Heo, S. H. *et al.* Composition change-driven texturing and doping in solution-processed SnSe thermoelectric thin films. *Nat. Commun.* **10**, 864 (2019).

21. Li, M. *et al.* Effect of the Annealing Atmosphere on Crystal Phase and Thermoelectric Properties of Copper Sulfide. *ACS Nano* **15**, 4967-4978 (2021).
